# Works on Operative and Mechanical Dentistry

**Published:** 1847-03

**Authors:** 


*Works on Operative and Mechanical Dentistry.*—That a work on operative and mechanical dentistry is much needed, all we think will admit, and it gives me pleasure to inform the readers of the Journal that Prof. A. Westcott, M. D., of the Baltimore College of Dental Surgery, has, in progress of preparation for the press, a work of this description. It is to be devoted to operative and mechanical dentistry, in their minutia of detail, giving, in the form of notes the principles involved in these departments, as also, the physical, mechanical and chemical properties of the various substances used by dentists, together with their application to science and art. It will also be illustrated with numerous engravings.

We have, also, been recently informed that Dr. W. H. Elliot, of Montreal, L. C., is also engaged in the preparation of a work on operative and mechanical dentistry, to be illustrated with engravings. That Dr. E. will produce a valuable work, we have abundant assurance from his contributions to the art, in the Journal. We have no doubt that both works will meet with a cordial reception from the members of the profession.—***Baltimore Ed.***

